# Naringin Reverses High-Cholesterol Diet-Induced Vascular Dysfunction and Oxidative Stress in Rats via Regulating LOX-1 and NADPH Oxidase Subunit Expression

**DOI:** 10.1155/2019/3708497

**Published:** 2019-10-24

**Authors:** Sirinat Pengnet, Sakdina Prommaouan, Phinsuda Sumarithum, Wachirawadee Malakul

**Affiliations:** ^1^Department of Physiology, Faculty of Medical Science, Naresuan University, Phitsanulok 65000, Thailand; ^2^Division of Physiology, School of Medical Sciences, University of Phayao, Phayao 56000, Thailand

## Abstract

Hypercholesterolaemia is associated with oxidative stress and endothelial dysfunction and leads to the development of atherosclerosis. Naringin exhibits cardiovascular protective and antioxidant properties. Therefore, the aim of this study was to assess the effect of naringin administration on vascular oxidative stress and endothelial dysfunction in hypercholesterolaemic rats and to elucidate its underlying mechanism. Sprague Dawley rats were fed a diet with 1.5% cholesterol (HCD) for 8 weeks to induce hypercholesterolaemia. Naringin (100 mg/kg body weight) was orally administrated to rats during the last 4 weeks of the diet treatment. After 8 weeks, the thoracic aorta was isolated to determine vascular function and nitric oxide (NO) levels. The aortic superoxide anion (O_2_^−^) level was detected using dihydroethidium (DHE) fluorescence staining. Protein expression of lectin-like oxidized low-density lipoprotein receptor-1 (LOX-1), nicotinamide adenine dinucleotide phosphate (NADPH) oxidase subunits, and inducible nitric oxide synthase (iNOS), as well as oxidative damage markers, was also evaluated in aortae. Naringin treatment of hypercholesterolaemic rats enhanced aortic NO levels, restored endothelium-dependent responses to acetylcholine (ACh), and reduced aortic O_2_^−^ levels. Furthermore, naringin treatment decreased LOX-1, NADPH oxidase subunits (p47^phox^, Nox2, and Nox4), and iNOS as well as oxidative damage markers (3-nitrotyrosine (3-NT) and 4-hydroxynonenal (4-HNE)) expression in aortic tissues from hypercholesterolaemic rats. These results demonstrate that naringin treatment improves endothelium dysfunction in hypercholesterolaemic rats, at least partially by decreasing oxidative stress via downregulation of LOX-1 and NADPH oxidase.

## 1. Introduction

Atherosclerotic cardiovascular disease is a major cause of morbidity and mortality worldwide [1]. Hypercholesterolaemia has been shown to be a major risk factor for atherosclerosis, associated with increasing oxidative stress and impaired endothelial function [2, 3]. The normal endothelium plays an important role in maintaining vascular homeostasis and regulating basal vascular tone through the production of several vasoactive substances, including nitric oxide (NO) and endothelin [4]. Dysregulation of endothelial function is thought to be one of the earliest manifestations of atherosclerosis and is characterized by reduced endothelium-dependent vasodilation, which is mainly mediated by NO [5].

Accumulating evidence has shown that reactive oxygen species (ROS) generated during hypercholesterolaemia are involved in key processes in the development and progression of atherosclerosis, including endothelial dysfunction and oxidative modification of low-density lipoprotein (oxLDL) [6, 7]. ROS triggers LDL cholesterol oxidation to form oxLDL, resulting in the upregulated expression of lectin-like oxidized low-density lipoprotein receptor-1 (LOX-1), an endothelial cell receptor for oxLDL [7]. Upregulated LOX-1 expression has been found in many pathological diseases, including hypercholesterolaemia [7]. Increased LOX-1 expression further stimulates the production of ROS, including superoxide (O_2_^−^) and hydrogen peroxide (H_2_O_2_), through the activation of *β*-nicotinamide adenine dinucleotide phosphate (NADPH) oxidase, thereby contributing to the enhancement of NO catabolism and the generation of peroxynitrite (ONOO^−^) [8, 9].

Vascular NADPH oxidase is one of the major sources of ROS generation in hypercholesterolaemia, atherosclerosis, hypertension, and diabetes and is often considered as a target for therapeutic intervention [10]. NADPH oxidase consists of multiple catalytic subunits, such as NOX (NOX1-5), as well as several regulatory subunits, such as p47^phox^, p67^phox^, and RAC [10]. Among the NOX catalytic subunits, Nox2, Nox1, and Nox4 have been detected in the cardiovascular system in many pathological diseases, such as hypercholesterolaemia, hypertension, and diabetes [11–13]. There is increasing evidence that ROS derived from NADPH oxidase not only decreases intracellular NO, but also increases LOX-1 expression, leading to further overproduction of ROS [8, 9, 14]. Excessive ROS attacks all macromolecules, including proteins, lipids, and nucleic acids, which is deleterious to various tissues and cells [15]. Therefore, natural products and compounds that suppress the expression of vascular NADPH oxidase and LOX-1 may offer therapeutic benefits to ameliorate the development of ROS-induced adverse vascular changes associated with hypercholesterolaemia.

Naringin is one of the flavonoids found in citrus fruit and has been reported to have antioxidant, antiapoptotic, anti-inflammatory, antihypercholesterolaemic, and cardioprotective properties [16–22]. Several studies have shown that it reduces the levels of plasma lipids and inflammatory markers and increases antioxidant activity in animal and human models of hyperlipidaemia [23, 24]. Additionally, naringin increased NO production which improved endothelial function in hypertensive rats [25]; normalized systolic blood pressure and improved vascular dysfunction and ventricular diastolic dysfunction in high-carbohydrate, high-fat diet-fed rats [26]; and ameliorated the impairment of endothelial function induced by high-fructose feeding [27]. Therefore, naringin is a useful compound for preventing the development and progression of atherosclerosis. However, its potential mechanisms in high-cholesterol diet-induced hypercholesterolaemic rats have not yet been clarified. We hypothesized that reduced ROS sources and decreased oxidative stress might be involved in improved endothelial function produced by naringin treatment in hypercholesterolaemic rats. Therefore, we sought to examine whether oral administration of naringin can decrease oxidative stress and improve endothelial function in hypercholesterolaemic rats.

## 2. Materials and Methods

### 2.1. Animal Experiments

The animal protocols for the study were approved by the Institutional Animal Care Committee of Naresuan University (ethical approval number: 5801001; approval date: January 28, 2015). Forty male Sprague Dawley (SD) rats weighing 180–200 g were purchased from the National Laboratory Animal Center at Salaya, Mahidol University, Thailand. The animals were housed in the animal room of the Center for Animal Research at Naresuan University at a controlled ambient temperature of 22 ± 1.0°C, with a 12-hour light/dark cycle. After an acclimatization period of 1 week, the rats were randomly divided into two groups: control and hypercholesterolaemic. Control rats were given a standard rat chow diet, while hypercholesterolaemic rats were given a high-cholesterol diet (HCD) for 8 weeks. The HCD was prepared according to a previous study [28] with slight modifications consisting of 1.5% cholesterol, 0.37% cholic acid, and 20% palm oil. After 4 weeks, the HCD induced hypercholesterolaemia in the rats, and then the control and hypercholesterolaemic groups were further divided into the following four subgroups: control (C, *n* = 10), control plus naringin (CN, *n* = 10), HCD (HC, *n* = 10), and HCD plus naringin (HCN, *n* = 10). Naringin (100 mg/kg) or vehicle (1% carboxymethylcellulose) was administered daily by oral gavage for the last 4 weeks of the study (from 5^th^ week to 8^th^ week of diet treatment). The dosage of naringin was used according to previous studies [26, 27], which reported that its administration restored impaired endothelial function in rats. At the end of the experiment, the rats were anaesthetized with an intraperitoneal injection of sodium pentobarbital (50 mg/kg/BW), and blood samples were collected via cardiac puncture for the analysis of lipid levels. Serum levels of total cholesterol (TC), triglyceride (TG), and high-density lipoprotein cholesterol (HDL-C) were measured using commercial test kits (Human, Wiesbaden, Germany). Low-density lipoprotein cholesterol (LDL-C) was calculated using the formula of Friedewald et al. [LDL-C=TC-HDL-C-(TG/5)] [29]. The atherogenic index of plasma (AIP) was calculated using the formula of Niroumand et al. [log (TG/HDL-C)] [30]. The atherogenic coefficient (AC) was calculated using the formula [(TC-HDL-C)/HDL-C]. The cardiac risk ratio (CRR) was calculated using the formula [TC/HDL-C].

### 2.2. Preparation of Isolated Aortic Rings

After the rats were euthanized, the descending thoracic aorta was dissected and cleaned of the surrounding connective tissues and superficial fat. One part of the isolated thoracic aorta was placed in Krebs bicarbonate solution (composition (mM): NaCl, 118.0; KCl, 4.7; MgSO_4_·7H_2_O, 1.2; KH_2_PO_4_, 1.1; NaHCO_3_, 25.0; D-glucose, 11.0; and CaCl_2_·2H_2_O, 2.5) for vasorelaxation response experiments, while another part was immediately frozen for nitrate and nitrite assays and western blot analysis.

### 2.3. Vascular Function Studies

The isolated thoracic aortae were cut into ring segments 2-3 mm in length, which were suspended between two stainless steel hooks linked to an isometric force transducer (AD Instruments Ltd, Australia) and connected to a Bridge Amplifier (PowerLab, AD Instruments Ltd., Australia) for isometric tension testing. Each aortic ring was placed in a 20 ml organ bath chamber containing Krebs bicarbonate buffer, maintained at 37°C with a pH of 7.4, and bubbled with a gas mixture (95% O_2_ and 5% CO_2_). Resting tensions of the aortic rings were maintained at 1 g, and the rings were allowed to equilibrate for 30 min. Bath contents were then replaced with an isotonic, 123 mM potassium physiological salt solution (KPSS) in which all the NaCl was replaced with KCl (123 mmol/L) to induce maximum contraction (KPSS_max_). The tissue was washed with fresh Krebs bicarbonate buffer, and the integrity of the endothelium was tested. For these tests, the aortic rings were then submaximally precontracted between 40% and 60% of the KPSS_max_ response with phenylephrine (PE, 1 nM–10 *μ*M). After the contractions were stabilized, acetylcholine (ACh, 10 *μ*M) was added to confirm the presence of endothelium.

Cumulative concentration-response curves to the endothelium-dependent relaxant ACh (1 nM–10 *μ*M) and the endothelium-independent relaxant sodium nitroprusside (SNP, 1 nM–10 *μ*M) were determined in vessels precontracted with PE (1 nM–10 *μ*M). The relaxation responses were continuously recorded until maximum relaxation was achieved.

### 2.4. Preparation of Aortic Homogenate

One of the aorta parts previously isolated was homogenized with RIPA lysis buffer (1% phosphatase and 1% protease inhibitor), centrifuged at 10,000 rpm for 10 min at 4°C to obtain the supernatants, and then stored at −80°C until analysis. The total protein level of the aortic homogenate was determined by using a bicinchoninic acid (BCA) assay and measuring the absorbance at 500 nm. Bovine serum albumin was used as the standard control.

### 2.5. Determination of Nitrate and Nitrite Levels in Aortic Tissue

Nitrate and nitrite levels in the aortic tissue were determined using a Nitrate/Nitrite Colorimetric Assay Kit (Cayman Chemical Company, Ann Arbor, MI, USA). Nitrate and nitrite levels were measured by using a microplate reader at a wavelength of 540 nm (Synergy™ HT, Winooski, USA).

### 2.6. Superoxide Detection

Superoxide generation in the aortic tissue was determined with dihydroethidium (DHE, 5 *μ*M). Aortic tissues were embedded in freezing medium, cut into 30 *μ*m thick sections, and incubated at 37°C protected from light for 30 min with the fluorescent probe DHE. In the presence of superoxide, DHE is oxidized to ethidium, which yields bright red fluorescence. After washing with PBS, the aortic sections were mounted and covered with a coverslip, and images were captured immediately with a fluorescent microscope at excitation and emission wavelengths of 520 and 610 nm, respectively. Superoxide generation was analysed with ImageJ software.

### 2.7. Western Blot Analysis

Protein expression levels of LOX-1, NADPH oxidase subunits (p47^phox^, Nox2, and Nox4), iNOS, and oxidative stress markers (3-NT and 4-HNE) in the aortic tissue were determined. Each sample of aortic homogenate (20 *μ*g proteins) was separated by using a sodium dodecyl sulphate-polyacrylamide gel electrophoresis (SDS-PAGE) system. After electrophoresis, the proteins were transferred to a polyvinylidene difluoride (PVDF) membrane. The blotted membranes were blocked with 5% skimmed milk for 1 hour at room temperature, followed by overnight incubation at 4°C with anti-LOX-1 (1 : 1000), anti-p47^phox^ (1 : 1000), anti-Nox4 (1 : 1000), anti-Nox2 (1 : 1000), anti-iNOS (1 : 1000), anti-3-NT (1 : 500), anti-4-HNE (1 :2000), and anti-*β*-actin (1 : 5000). The membranes were washed three times in Tris-buffered saline containing 0.2% Tween-20 (TBS-T) and then incubated with a goat anti-rabbit IgG-conjugated secondary antibody for 1 hr. After washing, specific proteins were detected using an enhanced chemiluminescence (ECL) system (Millipore, Billerica, MA, USA) and assayed by using Image Lab™ software (Bio-Rad Laboratories, Hercules, CA, USA). Data were expressed as percentages of the control.

### 2.8. Drugs and Chemicals

Naringin (purity > 90%), cholic acid, ACh, SNP, and PE were purchased from Sigma (St Louis, MO, USA). Primary antibodies against p47^phox^, Nox2, iNOS, and 3-NT were purchased from Merck Millipore (Darmstadt, Germany), *β*-actin was purchased from Cell Signaling Technology (Danvers, MA, USA), LOX-1 and Nox4 were purchased from Boster Biological Technology Co., Ltd. (Pleasanton, CA, USA), and 4-HNE was purchased from Abcam (Cambridge, MA, USA). Goat anti-rabbit IgG-conjugated secondary antibody was purchased from Santa Cruz Biotechnology (Dallas, TX, USA).

### 2.9. Statistical Analyses

The results were expressed as the mean ± SEM. Concentration-response curves were analysed using nonlinear regression to calculate the sensitivity of the concentration (pEC50). The maximum relaxation (*R*_max_) response to ACh and SNP was analysed as percentages of PE-induced contraction. The results were analysed by one-way analysis of variance (ANOVA) coupled with the Newman–Keuls multiple comparison test or Dunnet's test, where appropriate, using GraphPad Prism version 5. Statistical significance was indicated by a *p* value of less than 0.05.

## 3. Results

### 3.1. Body Weight, Lipid Levels, and Atherogenic Indices

The body weights and lipid profiles (TC, TG, LDL-C, and HDL-C) of the rats are shown in Table 1. There was no difference in initial body weight between the four groups of rats. At the end of the study, the final body weight of HCD-fed rats was significantly greater than that of control rats. Treatment with naringin for 4 weeks did not affect the body weight of normal rats, but significantly reduced the final weight of HCD-fed rats (Table 1). Feeding the rats with a HCD for 8 weeks caused a significant increase in TC, TG, and LDL-C and a decrease in HDL-C compared with control rats. Four weeks of treatment with naringin reversed the changes in the lipid profile induced by HCD in the HCN group (Table 1); however, the treatment did not change lipid levels in normal rats. As shown in Table 1, all atherogenic indices of the HC group were found to be increased compared with the control group. However, there were significant reductions (*p* < 0.05) in all atherogenic indices of the HCN group when compared with the HC group.

### 3.2. Effect of Naringin on Vascular Relaxation

**Figure 1:**
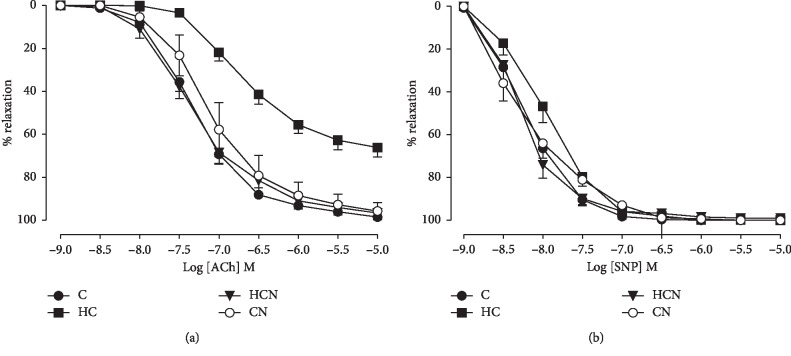
Cumulative concentration-response curves to ACh (a) and SNP (b) in aortic rings of control (C), hypercholesterolaemic (HC), HC + naringin (HCN), and C + naringin (CN) groups. The aortic rings were precontracted with PE. The pEC_50_ and *R*_max_ values presented in these graphs are shown in Table 2.

Both the sensitivity (pEC_50_) and maximum relaxation (*R*_max_) to ACh were significantly decreased in the aortic rings of hypercholesterolaemic rats compared with those of control rats (Figure 1(a) and Table 2), indicating that hypercholesterolaemia impaired endothelium-dependent relaxation in rat aortae. Treatment with naringin did not affect ACh-induced vasorelaxation in normal rats but increased the sensitivity and maximum relaxation to ACh in hypercholesterolaemic rats (Figure 1(a) and Table 2). In addition, there was no significant difference in the sensitivity and maximum relaxation to the endothelium-independent agonist SNP in the aortae of all groups (Figure 1(b)).

### 3.3. Effects of Naringin on Aortic Nitrate and Nitrite Levels

**Figure 2:**
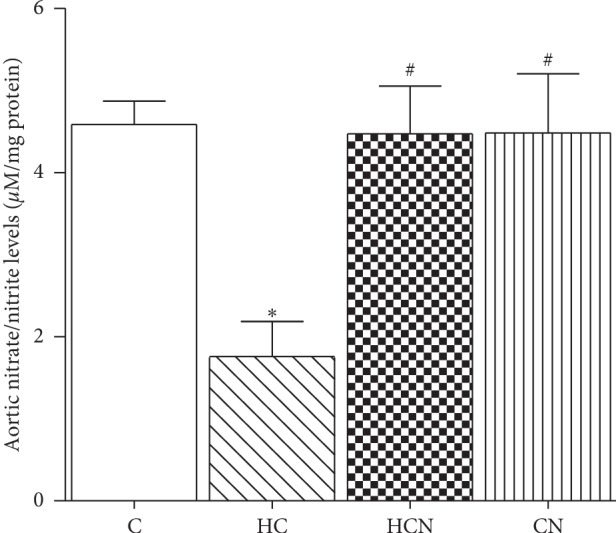
Levels of nitrate and nitrite from aortic tissues of control (C), hypercholesterolaemic (HC), HC + naringin (HCN), and C + naringin (CN) groups. The results are shown as mean ± SEM. *n* = 8–10 rats. ^*∗*^*p* < 0.05 vs. C group; ^#^*p* < 0.05 vs. HC group.

The levels of aortic nitrate and nitrite were decreased in hypercholesterolaemic rats when compared with the levels in the control rats (hypercholesterolaemic 1.76 ± 0.43 vs. control 4.59 ± 0.28 *μ*M/mg protein, *p* < 0.05) (Figure 2). Naringin treatment did not affect the levels of nitrate and nitrite in the aortae of control rats but significantly increased nitrate and nitrite levels in hypercholesterolaemic aortae to the levels observed in control rats. These results indicate that treatment with naringin enhances nitrate and nitrite levels in the aortic tissues of hypercholesterolaemic rats.

### 3.4. Effect of Naringin on Superoxide Production

**Figure 3:**
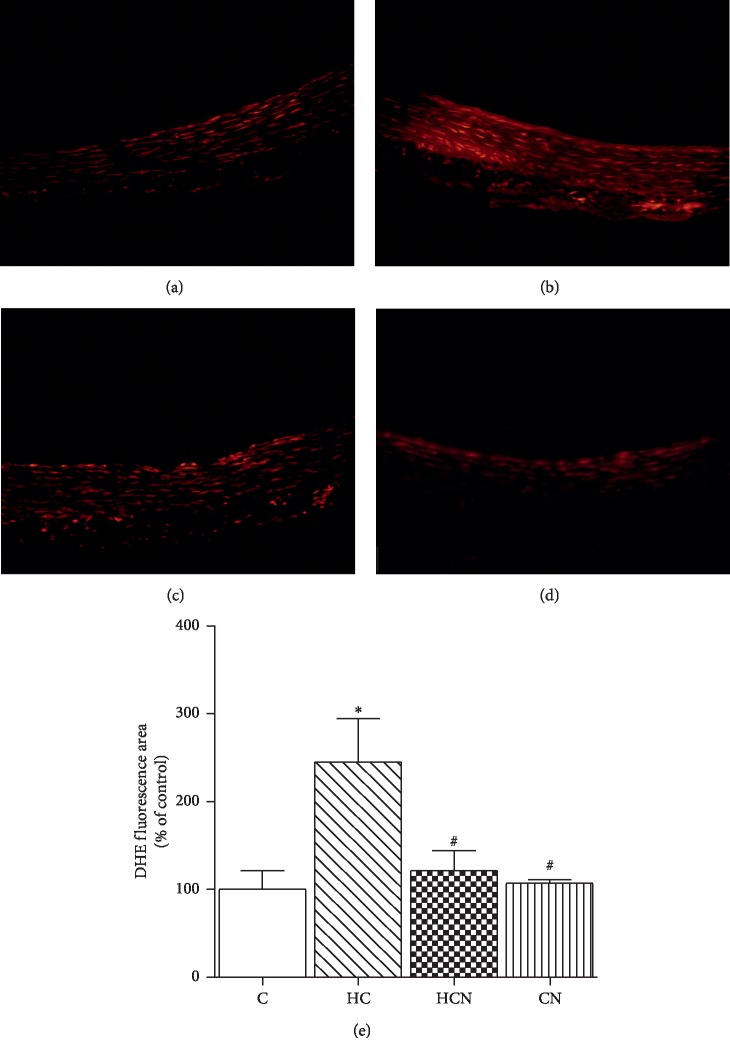
Detection of superoxide anions (O_2_^−^) generation in aorta. Representative photomicrographs of the staining (red signal) with the superoxide-detecting fluorescent probe, dihydroethidium (DHE) in aortic tissues from aortic tissues of control (C), hypercholesterolaemic (HC), HC + naringin (HCN), and C + naringin (CN) groups. Original magnification is 20x. Results are shown as mean ± SEM. *n* = 5 rats. ^*∗*^*p* < 0.05 vs. C group; ^#^*p* < 0.05 vs. HC group.

To evaluate superoxide anion levels in aortic tissue, DHE fluorescent staining was performed on aortic sections from each group; it revealed that superoxide levels in aortic tissues were significantly higher in HCD-fed rats than in control rats (Figure 3). However, DHE-derived signals were markedly attenuated in aortae from hypercholesterolaemic rats treated with naringin compared with untreated hypercholesterolaemic rats.

### 3.5. Effect of Naringin on LOX-1, NADPH Oxidase Subunits, iNOS, and Oxidative Stress Marker Expression

**Figure 4:**
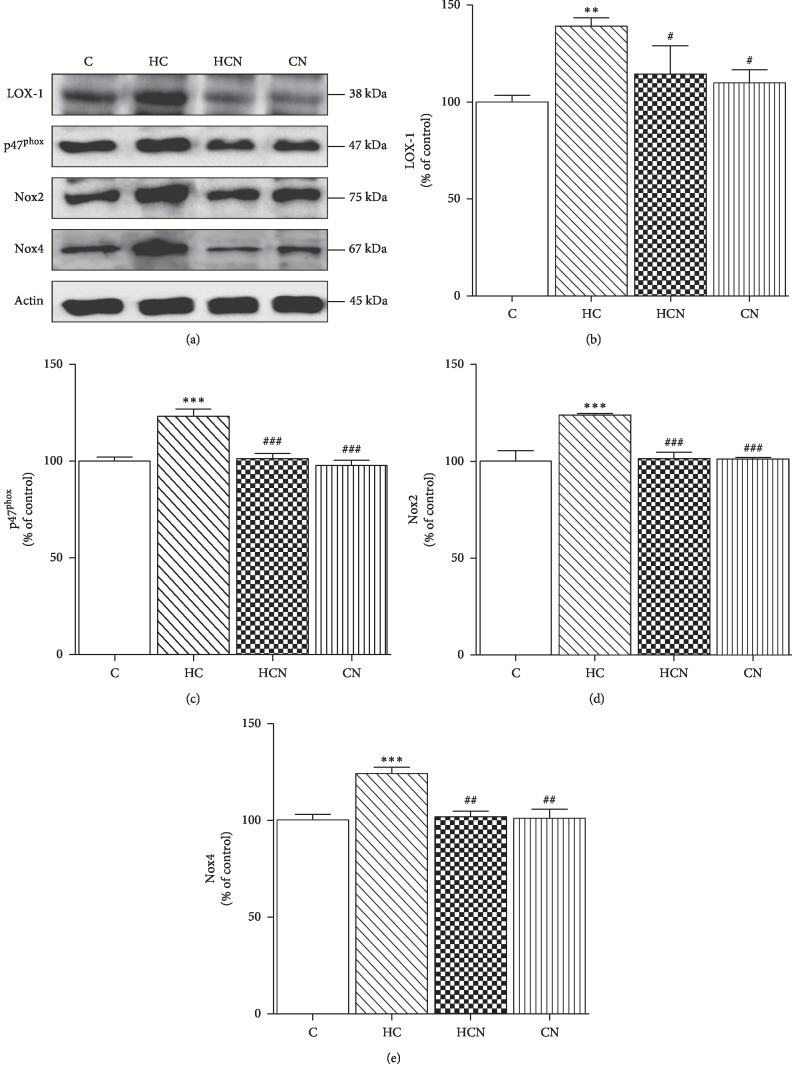
Western blot analysis of protein expression in aortae. Representative band of protein expression (a). Western blot of LOX-1 (b), p47^phox^ (c), Nox2 (d), and Nox4 (e) of control (C), hypercholesterolaemic (HC), HC + naringin (HCN), and C + naringin (CN) groups. The results are shown as mean ± SEM. *n* = 5 rats. ^*∗∗*^*p* < 0.01 and ^*∗∗∗*^*p* < 0.001 vs. C group; ^#^*p* < 0.05, ^##^*p* < 0.01, and ^###^*p* < 0.001 vs. HC group.

**Figure 5:**
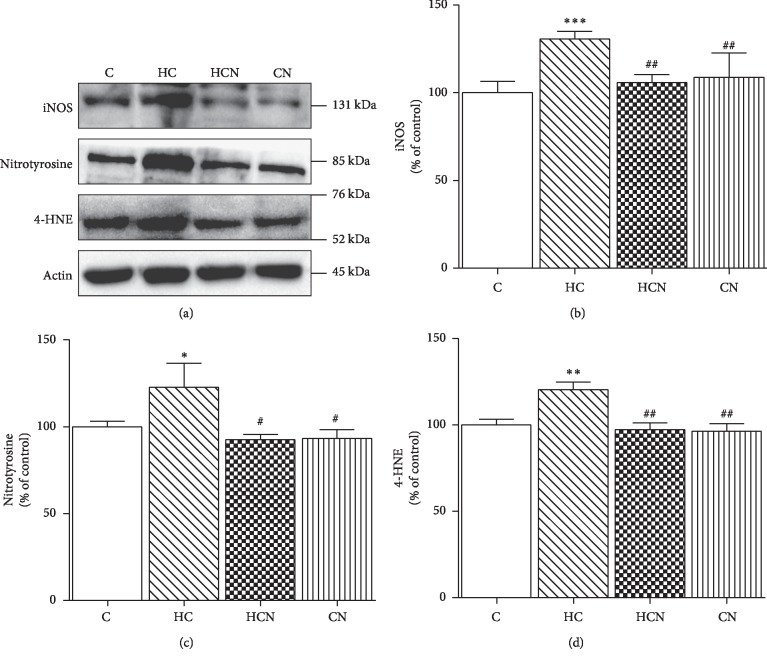
Western blot analysis of protein expression in aortae. Representative band of protein expression (a). Western blot of iNOS (b), 3-NT (c), and 4-HNE (d) of control C, hypercholesterolaemic (HC), HC + naringin (HCN), and C + naringin (CN) groups. The results are shown as mean ± SEM. *n* = 5 rats. ^*∗*^*p* < 0.05, ^*∗∗*^*p* < 0.01, and ^*∗∗∗*^*p* < 0.001 vs. C group; ^#^*p* < 0.05 and ^##^*p* < 0.01 vs. HC group.

Western blotting demonstrated that HCD-fed rats showed significantly increased expression of LOX-1, NADPH oxidase subunits (p47^phox^, Nox2, and Nox4), iNOS, and oxidative stress markers (3-NT and 4-HNE) in the aortic tissues as compared with control rats (Figures 4 and 5). Treatment of hypercholesterolaemic rats with naringin significantly decreased LOX-1, NADPH oxidase subunits (p47^phox^, Nox2, and Nox4), iNOS, and oxidative stress markers (3-NT and 4-HNE) expression in aortic tissues (Figures 4 and 5); however, this treatment did not alter these values in normal rats.

## 4. Discussion

Our results show that the treatment of hypercholesterolaemic rats with naringin for 4 weeks restores endothelium-dependent relaxation and NO levels in aortae. Furthermore, naringin treatment also decreased oxidative stress markers, including O_2_^−^, 4-HNE, and 3-NT, in aortic tissues of hypercholesterolaemic rats. In addition, we showed that the mechanisms associated with improvements in endothelial damage and oxidative stress involve the downregulation of LOX-1, NADPH oxidase subunits, and iNOS.

It is widely accepted that hypercholesterolaemia is one of the major risk factors for CVD and is characterized by abnormally high levels of serum total and LDL cholesterol [31]. It is well established that LDL cholesterol and especially its oxidized form (oxLDL) play important roles in impaired endothelial function and the pathogenesis of atherosclerosis [6, 32]. Numerous studies have shown that the consumption of a HCD causes serum lipid abnormalities and oxidative stress, thereby initiating endothelial dysfunction [33, 34]. Using standard diets supplemented with cholesterol and cholic acid to induce hypercholesterolaemia in rats in our study caused increased serum levels of TC, TG, and LDL-C and a decreased HDL-C level, indicating that a hypercholesterolaemic model was successfully induced by a HCD in the present study. Naringin was reported to exhibit hypocholesterolaemic activity by lowering cholesterol synthesis through the decreased activities of hepatic 3-hydroxy-3-methylglutaryl-CoA (HMG-CoA) reductase [19] and acyl-coenzyme A-cholesterol O-acyltransferase (ACAT) [35, 36]. Our data showed that after 4 weeks of administration of naringin, serum lipid levels from animals fed with high-cholesterol diets were found to be effectively decreased in HCD-fed rats. Additionally, atherogenic coefficient/indices, powerful indicators of the risk of cardiovascular diseases, were decreased in hypercholesterolaemic rats treated with naringin, indicating that naringin treatment reduced atherogenic risk in hypercholesterolaemia.

Endothelial dysfunction is considered an important early marker of atherosclerosis and characterized by impaired endothelium-dependent relaxation and reduced NO bioavailability [37]. The endothelium plays a crucial role in the regulation of tone by mainly synthesizing and releasing NO [4]. Several studies demonstrated that NO is the major mediator of endothelium-dependent relaxation of rat aortae and that mechanism of vasorelaxation is impaired in many pathological diseases, including hypercholesterolaemia and diabetes [5, 38, 39]. Endothelium-derived NO is synthesized by nitric oxide synthase (NOS) [37]. In vascular tissues, there are two principal types of NOS isoforms, endothelial NOS (eNOS) and inducible NOS (iNOS) [37]. eNOS has a protective vascular effect, whereas iNOS can produce a large amount of NO accompanied by increased generation of ROS, including OONO^−^, which are detrimental to vascular tissues [37]. Consistent with previous studies [40, 41], our results also demonstrate endothelial dysfunction in aortae of untreated hypercholesterolaemic rats, as shown by decreased ACh-induced endothelium-dependent vasorelaxation and reduced aortic content of the NO metabolites nitrite and nitrate. These findings indicated impaired endothelial function induced by hypercholesterolaemia related to reduced endothelium-derived NO levels. Previously, naringin was shown to restore the reduced ACh-induced vasorelaxation in the aortic rings of stroke-prone spontaneously hypertensive rats (SHRSP) [25] and of high-carbohydrate, high-fat diet-fed rats [26]. We found that naringin treatment for 4 weeks ameliorated the impairment of endothelium-dependent relaxation in hypercholesterolaemic rats, evidenced by an increase in the sensitivity and maximum relaxation of thoracic aortae in response to ACh. However, naringin treatment did not affect endothelium-independent relaxation of aortas in response to SNP. Furthermore, our results also demonstrated that naringin treatment increased the content of the NO metabolites nitrite and nitrate in aortic tissues of hypercholesterolaemic rats. These findings suggest that chronic treatment with naringin increases NO bioavailability and improves hypercholesterolaemia-induced endothelial dysfunction.

Considerable evidence indicates that overproduction of ROS under hypercholesterolaemic conditions causes the inactivation of NO and the development of endothelial dysfunction, as well as the oxidative modification of LDL [7, 42]. In vascular tissues, ROS are generated via several sources such as NADPH oxidase, xanthine oxidase, cyclooxygenase, uncoupled NOS, and mitochondria [10]. Several lines of evidence indicate that vascular NADPH oxidase plays a critical role in promoting ROS-induced vascular damage and atherogenesis [10]. NADPH oxidase consists of membrane-bound core catalytic subunits, such as Nox1–5 and Duox1-2, and cytosolic/regulatory subunits, such as p47^phox^, p67^phox^, and RAC [10]. Assembly of the membrane NADPH oxidase complex is activated by translocation of cytoplasmic subunits, such as the p47^phox^ and p67^phox^ subunits to the plasma membrane, which results in the generation of ROS, including O_2_^−^ and H_2_O_2_ [10]. There is evidence that Nox2 and Nox4 are the major isoforms of endothelial NADPH oxidase and are upregulated in various pathological diseases, including hyperlipidaemia [43, 44]. Our study showed that aortic expression of Nox2, Nox4, and p47^phox^ was increased in hypercholesterolaemic rats. Furthermore, in aortic tissues from the hypercholesterolaemic group incubated with DHE, there was an enhancement of red fluorescence, suggesting an increase in vascular O_2_^−^ levels. These findings were reversed by naringin treatment. In agreement with these results, similar changes in NOX subunits expression were associated with naringin treatment in other tissues of streptozotocin- (STZ-) induced diabetic models [45].

LOX-1, a receptor for oxLDL, has been reported to play a vital role in the activation of NADPH oxidase-derived ROS and is expressed on endothelial cells and smooth muscle cells [46]. Under hypercholesterolaemic conditions, elevated LDL-C is oxidized by ROS and turned into oxLDL [7]. The uptake of oxLDL into the endothelium via the receptor LOX-1 leads to the activation of NADPH oxidase followed by the quick enhancement of intracellular ROS, which causes macromolecular damage, including protein oxidation, lipid peroxidation, and endothelial dysfunction [8, 15]. ROS, particularly O_2_^−^, react with NO, thereby decreasing the availability of NO for vasorelaxation and generating OONO^−^, a highly reactive and cytotoxic molecule [15]. Furthermore, excessive NO produced by iNOS also promotes the formation of OONO^−^, which induces the nitration of protein-bound tyrosine residues, causing further oxidative damage to the vasculature [37, 47].

In an animal model of hyperlipidaemia, LOX-1 expression was found to be increased in the aorta of hyperlipidaemic mice [7]. In addition, hyperlipidaemia has been shown to trigger upregulation of 3-NT, a marker of peroxynitrite-induced protein nitration, and 4-HNE, an oxidized product from polyunsaturated fatty acid, in vascular tissues [44]. These observations were confirmed by the findings of our present study, which demonstrated that the aortic expressions of LOX-1, 3-NT, and 4-HNE, as well as iNOS expression, were enhanced in hypercholesterolaemic rats. These findings indicated the presence of oxidative damage in vascular tissues of hypercholesterolaemic rats.

Naringin has been demonstrated to exhibit strong antioxidant activities in scavenging free radicals [24, 48]. Previous studies showed that naringin decreased oxidative stress markers, such as malondialdehyde (MDA), and increased the activity of endogenous antioxidants, such as superoxide dismutase (SOD) and glutathione peroxidase (GSH-Px), in various tissues of high-fat diet-fed animals and in diabetic animals [24, 48]. Therefore, our study further examined the inhibitory effects of naringin on protein expression associated with oxidative stress. The present study, for the first time, showed the beneficial suppression of aortic expression of LOX-1 in hypercholesterolaemic rats by naringin, indicating that its antiatherosclerotic effect might be associated with a decrease in LOX-1 expression. Furthermore, naringin treatment reduced the expression of aortic oxidative stress markers 3-NT and 4-HNE, as well as the expression of iNOS in hypercholesterolaemic rats. All of the above results suggest that the beneficial effect of naringin on endothelial function in hypercholesterolaemia might be associated with the downregulation of LOX-1 and NADPH oxidase subunits. In addition, the decrease in both NADPH oxidase-derived O_2_^−^ and iNOS-derived NO by naringin treatment contributes to the prevention of OONO^−^ generation and NO inactivation, resulting in subsequent improvement of endothelial function.

## 5. Conclusion

The present study shows that naringin treatment for 4 weeks improves endothelial function in the aortae of hypercholesterolaemic rats. The mechanism related to the beneficial effects of naringin administration on hypercholesterolaemia is the reduction of oxidative stress in aortic tissues, possibly due to the downregulation of LOX-1 and the oxidative enzymes NADPH oxidase and iNOS. The reduction of ROS production leads to increased NO bioavailability and improved endothelium-dependent vasorelaxation in aortae of hypercholesterolaemic rats.

## Figures and Tables

**Table 1:** The parameters at the end of the experiment of control and hypercholesterolaemic rats with or without naringin treatment.

Parameter	C	HC	HCN	CN
Initial body weight (g)	195 ± 2	196 ± 7	195 ± 10	218 ± 1
Final body weight (g)	432 ± 5	479 ± 4^*∗∗*^	428 ± 9^##^	424 ± 12^##^
Serum TC (mg/dl)	67 ± 5	346 ± 17^*∗∗*^	164 ± 14^*∗∗*^^,##^	67 ± 1^##^
Serum TG (mg/dl)	39 ± 6	82 ± 5^*∗∗*^	48 ± 5^#^	38 ± 2^#^
Serum LDL-C (mg/dl)	28 ± 7	293 ± 8^*∗∗*^	113 ± 11^*∗∗*^^,##^	22 ± 2^##^
Serum HDL-C (mg/dl)	42 ± 1	25 ± 4^*∗∗*^	43 ± 3^##^	37 ± 2^#^
AC	0.75 ± 0.32	9.62 ± 1.82^*∗∗∗*^	4.69 ± 0.57^*∗*^^,##^	0.81 ± 0.10^###^
CRR	1.75 ± 0.32	10.62 ± 1.81^*∗∗∗*^	5.69 ± 0.57^*∗*^^,##^	1.81 ± 0.10^###^
AIP	0.01 ± 0.08	0.59 ± 0.15^*∗∗*^	0.30 ± 0.06^*∗*^^,#^	0.02 ± 0.03^##^

Results are shown as mean ± SEM for 8–10 rats. ^*∗*^*p* < 0.05, ^*∗∗*^*p* < 0.01, and ^*∗∗∗*^*p* < 0.001 vs. C group; ^#^*p* < 0.05, ^##^*p* < 0.01, and ^###^*p* < 0.001 vs. HC group. TC, total cholesterol; TG, triglyceride; LDL-C, low-density lipoprotein cholesterol; HDL-C, high-density lipoprotein cholesterol; AC, atherogenic coefficient; CRR, cardiac risk ratio; AIP, atherogenic index of plasma.

**Table 2:** A comparison of the sensitivity (pEC50) and maximum relaxation (*R*_max_) response to ACh and SNP in aortic rings from control and hypercholesterolaemic rats with or without naringin treatment for 4 weeks.

	ACh		SNP	
	pEC_50_	*R*_max_	pEC_50_	*R*_max_
C	7.32 ± 0.07	99 ± 1	8.50 ± 0.17	100 ± 0
HC	6.60 ± 0.07^*∗∗∗*^	65 ± 5^*∗∗∗*^	8.12 ± 0.13	100 ± 0
HCN	7.37 ± 0.10^###^	97 ± 1^###^	8.54 ± 0.24	100 ± 0
CN	7.13 ± 0.17^##^	96 ± 4^###^	8.46 ± 0.10	100 ± 0

Results are shown as the mean ± SEM. *n* = 8–10 rats. ^*∗∗∗*^*p* < 0.001 vs. C group; ^##^*p* < 0.01, ^###^*p* < 0.01 vs. HC group.

## Data Availability

The data used to support the findings of this study are included within the article.

## Abbreviations

TC: Total cholesterol
TG: Triglyceride
LDL-C: Low-density lipoprotein cholesterol
HDL-C: High-density lipoprotein cholesterol
AC: Atherogenic coefficient
CRR: Cardiac risk ratio
AIP: Atherogenic index of plasma
NO: Nitric oxide
eNOS: Endothelial NO synthase
ACh: Acetylcholine
SNP: Sodium nitroprusside
PE: Phenylephrine
LOX-1: Lectin-like oxidized low-density lipoprotein receptor-1
NADPH oxidase: Nicotinamide adenine dinucleotide phosphate oxidase
iNOS: Inducible nitric oxide synthase
3-NT: 3-Nitrotyrosine
4-HNE: 4-Hydroxynonenal
O_2_^−^: Superoxide anion.
